# Multi-Omics Pipeline and Omics-Integration Approach to Decipher Plant’s Abiotic Stress Tolerance Responses

**DOI:** 10.3390/genes14061281

**Published:** 2023-06-16

**Authors:** Rajib Roychowdhury, Soumya Prakash Das, Amber Gupta, Parul Parihar, Kottakota Chandrasekhar, Umakanta Sarker, Ajay Kumar, Devade Pandurang Ramrao, Chinta Sudhakar

**Affiliations:** 1 Department of Plant Pathology and Weed Research, Institute of Plant Protection, Agricultural Research Organization (ARO)—The Volcani Institute, Rishon Lezion 7505101, Israel; 2 School of Bioscience, Seacom Skills University, Bolpur 731236, West Bengal, India; 3 Dr. Vikram Sarabhai Institute of Cell and Molecular Biology, Faculty of Science, Maharaja Sayajirao University of Baroda, Vadodara 390002, Gujarat, India; 4 Department of Biotechnology and Bioscience, Banasthali Vidyapith, Banasthali 304022, Rajasthan, India; 5 Department of Plant Biochemistry and Biotechnology, Sri Krishnadevaraya College of Agricultural Sciences (SKCAS), Affiliated to Acharya N.G. Ranga Agricultural University (ANGRAU), Guntur 522034, Andhra Pradesh, India; 6 Department of Genetics and Plant Breeding, Faculty of Agriculture, Bangabandhu Sheikh Mujibur Rahman Agricultural University, Gazipur 1706, Bangladesh; 7 Department of Botany, Maharshi Vishwamitra (M.V.) College, Buxar 802102, Bihar, India; 8 Department of Biotechnology, Mizoram University, Pachhunga University College Campus, Aizawl 796001, Mizoram, India; 9 Plant Molecular Biology Laboratory, Department of Botany, Sri Krishnadevaraya University, Anantapur 515003, Andhra Pradesh, India

**Keywords:** abiotic stress, crop improvement, genomics, metabolomics, multi-omics, omics integration, phenomics, proteomics

## Abstract

The present day’s ongoing global warming and climate change adversely affect plants through imposing environmental (abiotic) stresses and disease pressure. The major abiotic factors such as drought, heat, cold, salinity, etc., hamper a plant’s innate growth and development, resulting in reduced yield and quality, with the possibility of undesired traits. In the 21st century, the advent of high-throughput sequencing tools, state-of-the-art biotechnological techniques and bioinformatic analyzing pipelines led to the easy characterization of plant traits for abiotic stress response and tolerance mechanisms by applying the ‘omics’ toolbox. Panomics pipeline including genomics, transcriptomics, proteomics, metabolomics, epigenomics, proteogenomics, interactomics, ionomics, phenomics, etc., have become very handy nowadays. This is important to produce climate-smart future crops with a proper understanding of the molecular mechanisms of abiotic stress responses by the plant’s genes, transcripts, proteins, epigenome, cellular metabolic circuits and resultant phenotype. Instead of mono-omics, two or more (hence ‘multi-omics’) integrated-omics approaches can decipher the plant’s abiotic stress tolerance response very well. Multi-omics-characterized plants can be used as potent genetic resources to incorporate into the future breeding program. For the practical utility of crop improvement, multi-omics approaches for particular abiotic stress tolerance can be combined with genome-assisted breeding (GAB) by being pyramided with improved crop yield, food quality and associated agronomic traits and can open a new era of omics-assisted breeding. Thus, multi-omics pipelines together are able to decipher molecular processes, biomarkers, targets for genetic engineering, regulatory networks and precision agriculture solutions for a crop’s variable abiotic stress tolerance to ensure food security under changing environmental circumstances.

## 1. Introduction

Stress could be defined as potentially unfavorable changes in environmental and/or biological factors that negatively affect plant growth, development and productivity [1]. Stress is categorized as abiotic and biotic. Living organisms such as fungi, bacteria, viruses, parasites, insects, weeds and native plants can induce biotic stress [2]. Whereas abiotic stress is caused by the effect of non-living environmental factors such as drought, salinity, temperature, cold, waterlogging, heavy metal, high light intensity, etc. [3], the impact of stresses on crop plants, either abiotic or biotic, is multidimensional and causes significant yield loss [4,5]. For the adaptation to environmental stresses, plants evolve several sophisticated mechanisms. Plants’ responsiveness to stress conditions entails sensing the stress, which activates a signal transduction pathway, activating stress-responsive genes via secondary messengers of signal transduction cascades and finally activating several stress-responsive genes and their products, which respond transcriptionally and translationally to the concerned abiotic stress. Due to the complex polygenic nature of the stress responses, there is a need to unravel the possible mechanisms of stress tolerance [6,7,8]. Deciphering the complex molecular regulatory network for a plant’s stress response requires cutting-edge genomic and molecular biology techniques such as high-throughput analysis of expressed sequence tags (EST), large-scale parallel analysis of gene expression, targeted or random mutagenesis and loss/gain-of-function or mutant complementation. These techniques have the potential to significantly improve our understanding of plants’ response to abiotic stress tolerance [9,10,11]. This technological advancement generates enormous amounts of information with thousands of new algorithms, tools and software, improvement in storage, processing and sharing of large datasets. “Omics” emerged as a new field that boosted the interaction of different modern biological approaches [12,13,14,15]. Omics is a term that refers to a set of molecular, system and computational biology tools that are used to assess the roles and interconnections of biological information in various clusters of life [16,17]. Multi-omics approaches include genomics, transcriptomics, proteomics, metabolomics, epigenomics, bioinformatics, proteogenomics, lipidomics, ionomics, interactomics and phenomics that provide a magnitude of data for understanding the physiological processes in plants under stress conditions and the tricky way to combat the harsh effect of those stresses [16,17,18,19]. Nevertheless, utilizing only a mono-omics approach does not provide sufficient knowledge to understand the complexity of plant responses under stress conditions. Therefore it is required to apply or integrate the multi-omics approaches for some promising output.

Innovative approaches that integrate the data from multi-omics layers, i.e., panomics, such as genomics, transcriptomics, proteomics and metabolomics hold great promise for enhancing crop improvement strategies [20]. By combining information from these different layers, it is possible to gain a comprehensive understanding of complex biological processes and identify key molecular players involved in a plant’s abiotic stress tolerance response. Several potential tools and approaches have been created for omics-data integration and interpretation due to the availability of multi-omics-pipeline-generated data collected from a wide range of samples and the introduction of high-throughput screening procedures by using data repositories and visualization portals [21,22]. While integrating multi-omics data, panomics can provide a more holistic and in-depth analysis of abiotic stress tolerance through data integration, system biological analytics, functional annotation and pathway analysis, data mining and machine learning for precise genomic prediction of crop germplasm [22]. It overcomes the difficulties encountered when integrating data from several different sources. Such integration of multi-omics data enables researchers to unravel intricate regulatory networks, identify candidate genes, proteins and metabolites, and discover potential biomarkers or targets for crop enhancement. This knowledge can aid in the development of stress-tolerant crop varieties through targeted breeding or genetic engineering approaches [21,22].

**Figure 1:**
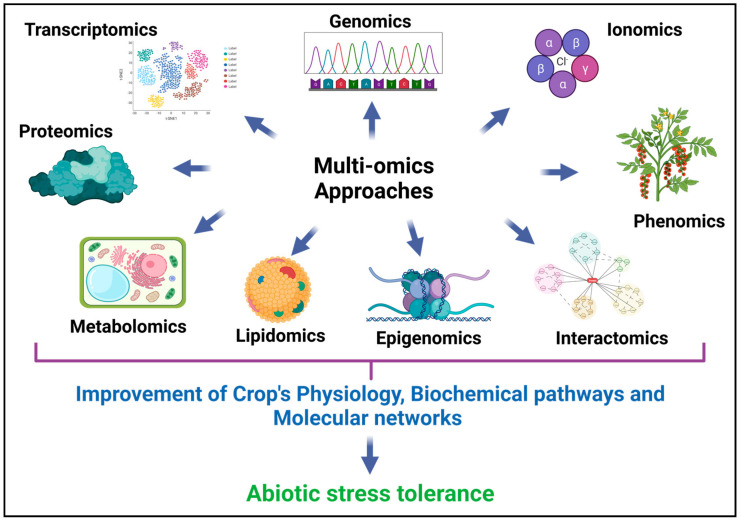
Integrative multi-omics approaches to confer abiotic stress tolerance in plants. The diagram was created using BioRender (https://biorender.com/) premium version.

To decipher crops’ abiotic stress tolerance response, both available marker-assisted breeding (MAB) and advanced multiomics-based analysis are complementary to each other, but omics-based tools provide a broader and more comprehensive understanding of plant biology of stress responses than MAB [23]. Omics technologies provide a comprehensive view of the entire plant system to study the complex interactions between genes, proteins and metabolites underlying molecular mechanisms of abiotic stress tolerance. It can identify novel genes and pathways associated with abiotic stress tolerance that may not have been previously recognized or targeted by marker-assisted breeding approaches [24]. In addition, omics pipelines enable high-throughput screening and selection of germplasm based on their genetic makeup, gene expression patterns, protein profiles or metabolite compositions which allows breeders to efficiently identify and select individuals with desirable stress tolerance properties [25]. Omics-based analysis can integrate data from various omics platforms, providing a systems-level understanding that enables the identification of key regulatory networks, biomarkers and candidate genes that can be targeted for breeding efforts. This holistic approach increases the chances of success in developing stress-tolerant crop varieties. The big data obtained from the multi-omics layers, combined with advanced bioinformatics and computational tools, can be used for predictive modelling and precision breeding by applying machine learning algorithms [26]. In the following sections, a detailed view on each omics approach and multi-omics integration has been discussed to bring a clear picture of abiotic stress tolerance mechanism in plants (Figure 1).

## 2. Genomics

**Figure 2:**
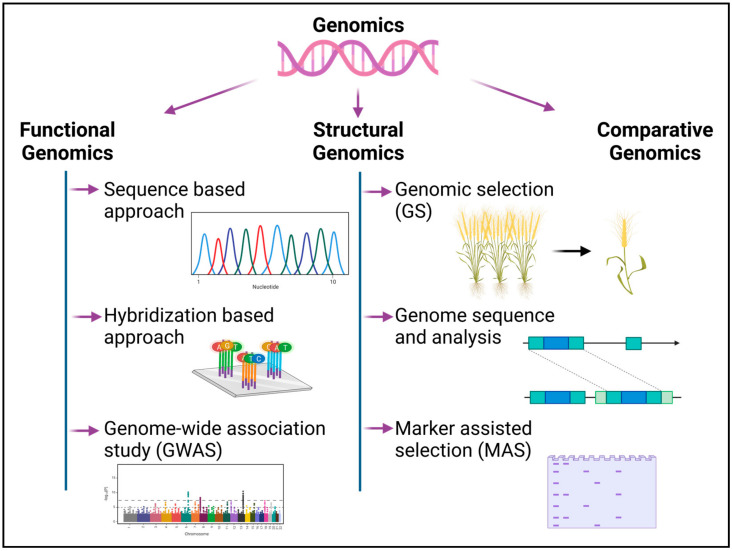
Different cohorts of genomics for crop assessment and improvement in relation to abiotic-stress tolerance response. The diagram was created using BioRender (https://biorender.com/) premium version.

A genome is an organism’s comprehensive collection of nucleic acids (DNA or RNA), which contains all of its genes. Genomic science or genomics is the study of the genome’s structure, function, evolution, mapping and modifications. Recent breakthroughs in molecular biological techniques have accelerated the pace of high-throughput genome sequencing, genomic characterization and gene expression analysis [27]. The technique of decoding the genome using high throughput next-generation sequencing (NGS) technology comprises the isolation of genomic DNA, the multiplication of DNA using polymerase chain reaction (PCR), the sequencing of the DNA and the assessment of the sequence’s integrity [28]. The sequencing and assembly of DNA, followed by the structural and functional annotation of the gene, permits large-scale investigations into the activities of genes and elucidates the interactions of gene products at the cellular and organismal levels [29]. The field of genomics has been discussed under three categories in the following sections (Figure 2):

### 2.1. Functional Genomics

Functional genomics analyzes the data generated by complete or partial genome sequencing to describe gene functions and interactions and employs two complementary approaches to the determination of individual genes, viz., forward and reverse genetics [30]. The forward genetic approach investigates a randomly obtained mutant of an interesting phenotype and identifies the responsible gene(s). On the other hand, the reverse genetic approach is the analysis of an organism’s phenotype by disruption of a known gene [31]. The technique of functional genomics helps in unravelling the gene interactions as well as the regulatory networks of genes and this technique employs the below-mentioned methodologies:

#### 2.1.1. Sequencing-Based Approaches

Exploring the expressed gene catalogue has been possible by analyzing the ESTs, i.e., the gene sequence produced from the cDNA clones by the single-pass method [32]. Utilizing the ESTs is a cost-effective as well as rapid method and is thus considered mainly in functional genomics studies. Deokar et al. [33] conducted an EST-based investigation in which they found differentially expressed genes (DEGs) in drought-susceptible and tolerant plants using the suppression subtraction hybridization (SSH) approach to build the analyzed plant’s EST library. After obtaining the ESTs, it gets submitted to the National Center for Biotechnological Information (NCBI), which serves as the source for EST sequencing to reveal the genes that are differentially expressed. Another sequence-based approach is Serial Analysis of Gene Expression (SAGE) which helps in quantifying the abundance of several transcripts together. In the SAGE, sequence tags of small stretch are joined and thereafter sequenced to analyze the gene expression [34] and the identification by these short tags depends on the presence of the EST database for a given species of consideration. SAGE technique is not very applicable to plant systems and thus has been modified as either SuperSAGE or DeepSAGE [35]. Similar to SAGE, the Massively Parallel Signature Sequencing (MPSS) approach has been used to study the long sequences with tags that are affixed to microbeads and then sequenced in parallel, allowing for the analysis of millions of transcripts simultaneously [36]. MPSS technique has high throughput and thus enables identifications to be performed with more specificity.

#### 2.1.2. Hybridization-Based Approaches

Another approach to studying the sequence is an array-based technique, where the hybridization of the DNA that has to be studied is carried out with the cDNA/oligonucleotide probes to assess the gene expression [37]. The limitation of this approach is that designing the probe requires the knowledge of the transcript either in the form of a sequence or a clone. Array-based data exist extensively for model plant species but there is a lack of data for the economically important crop plants and thus unravelling the stress responses utilizing these methods in crop plants becomes a difficult task.

#### 2.1.3. Expansions to Functional Genomics Approaches

Genome-wide association studies (GWAS) are an experimental and statistical examination of a large number of genetic variations across the genome in different organisms (or individuals) to determine whether any variant is related to a trait of interest. GWAS examines the entire genome to identify DNA variations related to the trait of interest [38]. GWAS has been successfully used in deciphering abiotic tolerance in rice [39], soybean [40], wheat [41], maize [42], sesame [43], barley [44], chickpea [45], rapeseed [46], cotton [47,48,49] and sorghum [50,51]. The primary goal of GWAS is to identify genomic areas linked with agronomic or morphological features or any phenotypes that can be markers, genes or quantitative trait loci (QTL) for gene discovery, introgressive hybridization and MAB [52] (Table 1). Advances in genomics and phenomics have resulted in a more precise and comprehensive characterization of QTLs, often referred to as QTLomes [53]. Presently, the QTLome concept is being utilized in specific QTL alleles associated with traits. In addition, numerous statistical methods, such as meta-QTL analysis, have aided in the collection of QTL data from various studies on the same linkage to pinpoint the precise QTL region [41]. This meta-analysis has been applied to study important crop plants such as wheat, soybean, etc. Utilizing the meta-analysis study, Ha et al. [54] identified loci for salt tolerance in soybean on chromosome 3 and used simple sequence repeat (SSR) and single nucleotide polymorphism (SNP) markers to analyze the RIL population (PI 483463 Hutcheson). Sheoran et al. [55] identify the candidate genes of maize for abiotic stress tolerance and utilization in future breeding for crop improvement. Moreover, such meta-QTL analysis also helped to screen the genomic loci of rice for salinity and drought tolerance in different growth and developmental phases—seedling and flowering stages [56,57].

In the 21st century, gene editing has emerged very alarmingly for functional characterization and validation of newly identified genes or genetic regions associated with stress-responsive genes in plants [58]. Success in manipulating a specific gene with a respective function may be achieved by the use of the clustered regularly interspaced short palindromic repeat (CRISPR)—Cas (CRISPR-associated system), which is a more concise, less labor-intensive alternative to traditional methods such as meganucleases (MNs), zinc-finger nucleases (ZFNs), transcription-activator-like effector nucleases (TALENs) [59]. The CRISPR-Cas system of gene editing approach becomes very efficient to characterize the functionality of plant-responsive genes for drought [60,61], salinity [62,63], heat [64] and cold stress [65].

### 2.2. Structural Genomics

Functional genomics is concerned with the function of genes and their interactions. Structural genomics is concerned with determining the three-dimensional structure of genes to identify, locate and determine their order along the chromosome [60]. Functional and structural genomics studies corroborate the intricate links between sequence and structure, ultimately offering the complete genome, which can aid in the understanding of a wide range of biological issues [66].

#### 2.2.1. Genomic Selection (GS)

Genomic selection (GS) permits the quick selection of better genotypes by utilizing high-density markers distributed across the genome [67]. GS is a novel strategy for optimizing quantitative characteristics; it utilizes marker and phenotypic data from observed populations to assess the impact of all loci [68,69]. Generally, the method of genomic selection relies on two types of datasets: a training set and a validation set [70,71]. The training data set is the reference population and is used for the estimation of marker effects, whereas the validation set possesses the selected candidates that have been genotyped [72].

#### 2.2.2. Genome Sequencing and Mapping

DNA sequencing has provided many details on the sequence, including the whole genome. There are several platforms, such as Roche 454GS FLX Titanium or Illumina Solexa Genome Analyzer, that are said to be NGS platforms that have helped in reducing the sequencing cost as well as time in comparison to conventional sequencing methods such as the Sanger method [73]. The sequencing method helps in developing improved varieties of crops by sequencing and resequencing processes. To date, the genomic sequence for several crop plants such as rice, wheat, maize, sorghum, soybean and tomato has been published. Apart from these crop plants, the sequence of model plants such as *Arabidopsis thaliana* and *Brachypodium distachyon* has also been published [74]. Genome sequencing provides detailed data on the features of genomes (coding as well as non-coding genes), GC content, repetitive elements as well as regulatory sequences [75]. Although genome sequencing provides important details for improving crops using molecular breeding, its usefulness is limited to species that have a smaller genome. To facilitate a complex genome study, another technology that is chromosome-specific has helped in developing Bacterial Artificial Chromosome (BAC) libraries to help in studying the complex genome. Mapping of 1 Gb chromosome of wheat has been possible with the help of the chromosome-by-chromosome approach only [76]. Mapping compiles genetic mappings into physical contigs as well as providing a framework for the assembly of sequences into the whole genome, and in the absence of a reference genome sequence, this BAC-end shotgun sequence gives details of genome evolution as well as structure [77,78]. Another interesting method for developing a whole genome sequence has been carried out by detecting the QTL using a methodology named QTL-seq. A QTL is a polymorphic locus that differentially affects the trait. QTL mapping is the technique of utilizing DNA markers to generate linkage maps and identify genomic areas linked with certain characteristics. QTL mapping is used to characterize the organization and evolution of the chromosomes [79,80]. So far, several QTLs have been reported for tolerance to drought [81,82], salinity [39,83,84,85,86], heat [87,88], cold [46,89], etc. (Table 1).

**Table 1:** Important QTLs/markers identified for abiotic stress response in field crops.

Plants	QTLs/Markers	Chr. Location	Methods Used	Abiotic Stresses	References
Rice	OsHKT1;1	Chr 1	GWAS	Salinity	[39]
	qWUE.STI6	Chr 6	Linkage mapping	Drought	[80]
	Saltol	Chr 1	Linkage mapping	Salinity	[87]
	qCTBB2 qCTBB3	Chr 2 Chr 3	Linkage mapping	Cold	[89]
	qSTS4	Chr 4	QTL-seq	Salinity	[90]
Soybean	AX-93897192	Chr 19	GWAS	Phosphorus efficiency	[40]
	qGI10-1	Chr 10	GWAS	Drought	[79]
	qSFT_3-38, qSFT_7-3	Chr 3 Chr 7	Linkage mapping	Flooding	[91]
	qST6 qST10	Chr 6 Chr 10	Genotype-based sequencing (GBS)	Salinity	[92]
Wheat	MQTL1D.4 MQTL2D.5 MQTL3A.1	Chr 1D Chr 2D Chr 3A	MetaQTL	Drought stress Heat, Salinity Waterlogging	[41]
	QNa.asl-2A	Chr 2A	Genotype-based sequencing (GBS)	Salinity	[81]
	YIELD_MQTL4B.2_D	Chr 4B	MetaQTL	Heat, Drought	[85]
	qWMs108_7-1	Chr 7-3A	Linkage mapping	Drought	[93]
	QSpad3.ua-1D.5	Chr 1D	GWAS	Waterlogging	[94]
	QMrl3B(T2|T1)	Chr 3B	Linkage mapping	Salinity	[95]
Maize	*Zm*00001eb013650	Chr 1-10	GWAS + RNAseq	Salinity	[42]
	qPOD2b	Chr 2	Genome-Wide Association Study (GWAS)	Cold	[96]
Rapeseed	SA07_23415428	Chr SA07	GWAS	Freezing	[45]
	qDSI_SL-11-3 qDSI_RL-11-1 qDSI_RL-11-4 qDSI_SL11-3	Chr C01	Linkage mapping	Drought, Freezing	[97]
	qRRL.A3b	Chr A03	Linkage mapping	Waterlogging	[98]
Barley	QcRWC.3H_2.1 QcWC.3H_1 3H	Chr 3H	Linkage mapping	Drought	[76]
	HORVU2Hr1G111780.3	Chr 2H	Linkage mapping	Salinity	[82]
	qSLS-4	Chr 4H	Linkage mapping	Salinity	[88]
	QBIO.2H	Chr 2H	GWAS	Waterlogging	[99]
Cotton	qtlCSI01	Chr 3	Composite interval mapping	Drought	[47]
	qGR-Chr4-3, qFER-Chr12-3, qFER-Chr15-1	Chr 4 Chr 12 Chr 15	Linkage mapping	Salinity	[48]
	qEC_A02_ck qFW_A06_150.1	Chr 2 Chr 6	Genotyping by Sequencing (GBS)	Salinity	[49]
	qFSHa1	Chr 15	Composite interval mapping	Heat	[86]
Sorghum	qPH-6 qMC2-9	Chr 6 Chr 9	Genotype-based sequencing (GBS)	Excess soil nitrogen	[50]
	qTB45_4.S	Chr 4	Linkage mapping	Salinity	[51]

#### 2.2.3. Molecular Marker Resources

DNA markers are short areas of DNA sequences that have the ability to identify variations in a population’s DNA or polymorphisms (base deletion, insertion and substitution), including base deletions, insertions and substitutions. DNA markers are also known as genetic markers [11]. Molecular markers aid in tagging genomic traits such as pathogen resistance, abiotic stress tolerance, quantitative analysis, etc. Recent advancement in this resource has provided a new horizon for the genetic improvement of traits for stresses such as drought, salt, etc. [11]. To date, several molecular markers have been reported that help in identifying polymorphism in plants, and these markers include random amplified polymorphic DNA (RAPD), restriction fragment length polymorphism (RFLP), amplified fragment length polymorphism (AFLP), SNP, SSR and sequence-tagged sites (STS) [11,100]. Restriction fragment length polymorphism (RFLP) is the most basic marker that helps in identifying the polymorphism arising due to mutation or deletion/insertion leading to either formation/deletion of endonuclease recognition sites in restriction fragment length [101]. Another marker, RAPD, which is generated via random primers, identifies complementary sites at a short distance within the genome, while AFLP combines the restriction digestion as well as the PCR amplification and thus helps in identifying the linkages [102]. The SSR or microsatellite markers are tandem repeats of short mono-, di-, tri- and tetra-nucleotides and help in measuring the genetic diversity among species and also differentiate alleles that are homozygotic and heterozygotic between the lines from the same origin [103]. The SNPs are used for the characterization of germplasm as well as gene mapping. Due to their high abundance, codominance and sequence tagging they help in understanding complex traits utilizing microarrays such as Affymetrix GeneChip. Marker-assisted selection (MAS) is a genomic approach to identifying and breeding associated allelic markers [104]. During the process of marker-assisted selection, a characteristic of interest is chosen on the basis of a marker that has been associated with a particular or multiple abiotic stress [105,106]. Previously, success in MAS for abiotic stress tolerance was lagging due to the limited availability of genomic data. Genome databases and datasets that are very valuable in the construction of SSRs and SNP markers have been produced thanks to recent improvements in high-throughput DNA sequencing and genotyping technology [107]. Such availability of various high-throughput molecular markers and genome sequencing technologies leads to genomics-assisted breeding [108] and SNP difference-based haplotype mapping [109] to improve crops with stress tolerance properties.

### 2.3. Comparative Genomics

Comparative genomics is the science of comparing entire genomes or parts of genomes to find out basic biological similarities and differences as well as investigating evolutionary relationships between organisms [110]. Comparative genomics compares biological sequences by aligning them and detecting conserved sequences. Thus, studies of comparative genomics have revealed considerable synteny in related species [111]. Moreover, as comparative genomics can detect small-scale changes within different genomes, comparative studies of protein-coding regions and their consequences on protein structure and function identify important regulatory elements within DNA [112]. Comparative genomics gave rise to the “genome zipper” concept that helps in determining the virtual gene order within the partially sequenced genome. Genome zipper links the annotated and fully sequenced genome of sorghum, *Brachypodium* and rice with the data of less-studied species to predict the gene order and organization of the gene [113,114].

## 3. Transcriptomics

The whole collection of transcripts that are present in a cell or organism is referred to as the transcriptome, and the study of the transcriptome is referred to as transcriptomics [115]. It mainly helps in finding gene transcripts or RNA that are associated with a plant’s phenotypic expression under different environmental conditions [116]. A variety of methods, including DNA microarrays, SAGE or high-throughput technologies relying on NGS, may be used in the process of conducting a transcriptome study such as RNA sequencing (RNAseq) and digital gene expression (DGE) [117,118,119,120,121]. To date, transcriptomic analysis has identified many stress-responsive genes, and their mode of expression under abiotic stress conditions in many plants including wheat [122], maize [123], rice [124], barley [125], sorghum [126], cotton [127] and soybean [128]. Moreover, transcriptome analysis in tomatoes has revealed the discovery of regulators of SGA pathways such as GLYCOALKALOID METABOLISM (GAME) 9, also called JRE4, an AP2/ERF transcription factor in response to various abiotic stresses [129]. Meta-transcriptome analysis in rice revealed the expression of 6956 abiotic stress tolerance (*ASTR*) genes, some transcription factors (TFs) and a few functional modules such as cis-motifs. Out of the expressed *ASTR* genes, 1% were found to be colocalized within the trait-associated QTL and over 65% of the genes in the tolerant genotypes showed differential expression under saline, high temperature and drought stress environments [130]. Similar to this, Azzouz-Olden et al. with the help of transcriptome analysis and RNA sequencing data, reported two sorghum lines, viz., SC56 (drought-tolerant) and Tx7000 (drought-sensitive), the former showed overexpression of antioxidant genes such as *SOD1*, *SOD2*, *VTC1*, *MDAR1*, *MSRB2*, *ABC1K1*, regulatory factors such as CIPK1 and CRK7 and repressors of senescence, i.e., *SAUL1* [131]. Initially, microarray experiments detected the co-expressed genes during abiotic stress conditions. However, the most significant disadvantage of using microarray analysis is that information about the transcripts cannot be obtained from the genome as a whole. As a consequence of this, the studies using microarrays are unable to fully decode the regulatory gene networks that are involved in the abiotic stress response of plants. Fortunately, recent developments in molecular methods have made it possible to construct high-throughput procedures that are based on sequence-based methodologies [132]. The most widely used method for analyzing transcriptomes is called RNAseq. This is because it provides comprehensive coverage of the genome and ubiquitous expression of transcripts [133]. RNAseq detects DEG and consequently deciphers the regulatory mechanism of plant abiotic stress tolerance. Tiwari et al. [134] carried out transcriptome analysis using Illumina NextSeq500, and the outputs exhibited DEGs in different parts of plants. For example, there was up-regulation of 761, 572 and 688 DEGs and down-regulation of 280, 292 and 230 DEGs in the shoot, root and stolon, respectively. Moreover, fewer DEGs such as Myb-like DNA-binding protein, WRKY transcription factor 16, glutaredoxin family protein, malate synthase, CLE7, 2-oxoglutarate-dependent dioxygenase FLOWERING LOCUS T BTB/POZ domain-containing protein, F-box family protein and aquaporin TIP1;3 responsive to N-deficiency were also found to be expressed [134]. A list of potent genes associated with plants’ abiotic stress response is presented in Table 2 and those are highly important for further transcriptome analysis under varied and combined stress environments.

## 4. Proteomics

The study and characterization of the whole sequence of proteins that are present in a cell, organ or species at a particular point in time are referred to as proteomics. Changes in plant proteomes are an extremely significant topic to research since proteins are the primary key regulators of the plant’s stress response. Various stages, such as development, cellular differentiation and the cell cycle, as well as distinct environmental factors, such as abiotic stressors, may cause the same genes to express themselves in a variety of different ways [155]. As a result, various sets of proteins are produced by cells depending on the environment they are in. Because of this, some proteins might be regarded to be unique biomarkers for certain environmental factors such as abiotic stressors [156]. Therefore, proteomics research may lead to the discovery of these putative protein markers and variations in their abundance may be correlated with quantitative shifts in specific physiological indicators related to a person’s capacity to withstand stress [157]. Under environmental stresses, proteomics allows for the detection and characterization of proteins, as well as their activity profiles, post-translational modifications (PTMs) and protein–protein interactions [158,159,160,161,162]. A fundamental framework for a comparative analysis of the drought stress proteome changes in cereal crops such as wheat, rice, maize, barley, sorghum and pearl millet has been provided by a comprehensive study of the existing proteomics data sets [161]. The two most common laboratory techniques are used extensively in proteomics studies—protein electrophoresis and protein identification using mass spectrometry. Conventional gel-based protein electrophoresis methods include techniques such as two-dimensional electrophoresis (2-DE) and Difference In-Gel Electrophoresis (DIGE) [160,162]. Gel-based methods’ primary benefits consist of their ease of use, repeatability, broad molecular mass coverage and sensitivity to the detection of post-translational changes [163]. However, the 2-DE method possesses some inherent limitations such as reproducibility, detection of less abundant and hydrophobic proteins, identification of basic proteins, co-migration of proteins and presence of exceedingly large or small proteins [164]. Multi-dimensional Protein Identification Technology (MudPIT), a non-gel method of both qualitative and quantitative proteomic analyses has been popularly used to overcome the limitations of 2-DE. The mass spectrometry (MS) approach includes techniques such as liquid chromatography-MS (LC-MS/MS), Ion Trap–MS (IT-MS) and matrix-assisted laser desorption/ionization–MS (MALDI-MS). Fluorophore-tagged protein immune-precipitation and label-free MS-based quantification approaches have been developed [165] to achieve a higher level of precision in the identification of low-abundance signalling and regulatory protein complexes. In addition to this, Laser-Capture Micro-dissection, commonly known as LCM, has been used for the identification of tissue- and cell-specific proteins that play a crucial role in the response of crops to environmental stresses [166]. In the process of regulating a plant’s response to environmental stress, post-translational protein modifications, such as phosphorylation, redox and glycosylation, perform an essential role. The technique of phosphoproteomics, which is based on mass spectrometry, is an extremely useful instrument for determining the in vivo kinase activity of proteins. Immobilized metal affinity chromatography (IMAC) and immunoprecipitation utilizing antibodies towards phosphorylated amino acids study are two more methods that have allowed the discovery of hundreds of novel in vivo phosphorylation sites [163]. Stable isotope labelling by/with amino acids in cell culture (SILAC) and isobaric tags for absolute and relative quantification (iTRAQ) seem to be more sophisticated techniques that may monitor changes in the specific kinase domain. So far, various proteomic studies revealed abiotic stress tolerance of the plant, such as in drought [167,168], heat [158,169], chilling [159,170], salinity [156,171] and waterlogging [172,173]. Comparative proteome analysis for *Medicago sativa* cv. Zhongmu-1 and *Medicago truncatula* cv. Jemalong A17 roots were experimented with by using 2D gel electrophoresis and mass spectrometry under salt stress and revealed the abundance of 93 and 30 proteins was affected, while tandem spectrometry revealed expression of 60 and 26 proteins, respective cultivars and these proteins have been supposed to play important role in salinity stress [174]. Quantitative proteome analysis by Zhu et al. [175], revealed the expression of 179 salt–alkali responsive proteins and it was suggested that these proteins have a role in the tri-carboxylic acid (TCA) cycle, oxidative phosphorylation, glycolysis, sucrose metabolism as well as being involved in reactive oxygen species (ROS) homeostasis. Another, study considering the comparative proteome analysis revealed the expression of 37 proteins under salinity stress and these proteins were identified with their role in the process of photosynthesis, stress response and phytohormone biosynthesis [176].

## 5. Bioinformatics

Bioinformatics is a wide multidisciplinary field that encompasses both theoretical and practical methods to comprehend, generate, analyze and disseminate biological information. Bioinformatics is the first link between biological data and the application of computational methods [177]. To analyze and alter resources from databases [178], computational tools and methodologies provided by bioinformatics may be used. This may result in the production of novel findings or hypotheses, even though these may need proper evaluation [179]. The Integrative Omics–Metabolic Analysis (IOMA) platform was developed to combine proteomic data with cellular metabolic information. Thus, with time, the field of bioinformatics has evolved and provides the platform for interactive “Omics” technologies [180]. Several studies have involved bioinformatics tools to understand the overall underlying mechanism under abiotic stress conditions [178,180,181]. Although bioinformatics provides the platform for integrating the omics approaches by evolving with more and more novel technologies to provide in-depth knowledge of the complex data set to understand the individual analyses as well as a comparative analysis [182]. For example, the modern genome editing tool CRISPR-Cas9 approach can be utilized for analyzing different abiotic stress conditions and improving crop plants but it requires appropriate development of genomics as well as bioinformatics pipelines to provide more detailed information on how this process can be done [62,183]. A number of software packages such as E-CRISP, TIDE, CHOPCHOP and CCTop have been developed to envisage and select CRISPR-Cas9 for genome editing [62,184]. Recently, for agricultural communities, the gene ontology (GO)-analysis toolkit earlier called AgriGO has been rereleased with an updated tool under the name AgriGO2.0. This toolkit provides additional bioinformatics analysis such as singular enrichment analysis (SEA), transfer IDs by BLAST (BLAST4ID), parametric analysis of gene set enrichment (PAGE), etc. [185].

## 6. Epigenetics-Aided Epigenomics

The term ‘epigenetics’ was originally coined by Waddington in the middle of the 20th century by combining genetics and epigenesis to explain the phenotypic features of the plant due to the genetic interactions and its products [186]. In general, epigenetics refers to the non-heritable/heritable changes, cell division (mitotic or meiotic), methylation pattern of cytosine (C) nucleotide in DNA and histone protein modification which is concerned with various epialleles in the genomic region [187]. Epialleles have been reported as a major factor for phenotypic diversity [188]. Post-translational modifications are another major player that affects gene expression and makes changes in phenotypes. The study of phenotypes in a species due to epigenetics modification refers to epigenomics [189]. Acetylation, sumoylation, phosphorylation, methylation, ubiquitination, glycosylation, carbonylation and ADP ribosylation are the major post-translational modifications to modify the histone tails [190]. Apart from post-translational modification, chromatin remodelling is another factor that contributes to epigenetic modification in crop species. In addition to the modification, it is related to some sort of trans-generational inheritance, which affects the accessibility of DNA transcription factors (TFs) [191]. Simply, the transmission of epigenetic modification traits from one generation to the next is known as “transgenerational memory” which is independent of DNA sequences [192]. Alternatively, epigenetics also refer to the changes in gene activity without any alteration in DNA sequence. Epigenetic mechanisms result in the modification of chromatin structure which regulates mRNA accumulation at the transcriptional level [193]. Recent research showed that epigenetic mechanisms play a critical role in the regulation of plant genes’ expression under various abiotic stress conditions [194,195]. Changes in environmental factors such as temperature, day length, ultraviolet (UV) radiation, availability of water and soil salinity in plants lead to modifications in the (de)methylation pattern of the coding regions in many stress-responsive genes which consequently regulate their expression [193,194,195,196]. Nevertheless, in addition to DNA methylation, recent evidence showed that histone modification, small regulatory RNA (sRNA) and long non-coding RNA (lncRNA) associated regulatory pathways are other adaptive mechanisms that regulate gene expression under stress conditions [197]. In *A. thaliana*, four DNA methylases have been characterized: ROS 1 (REPRESSOR OF SILENCING 1), DME (DEMETER), DML2 (DEMETER LIKE 2) and DML3 (DEMETER LIKE 3). The DNA demethylase (glycosylase) replaces the 5- methylated cytosine with unmethylated cytosine through a base excision repair mechanism [198]. AtDME has been reported as an epigenetic regulator that is essential for maternal allelic expression of the MEA (MEDEA) gene which encodes a repressive H3K27 methyltransferase, in the embryo and endosperm central cell [199]. DEMETER LIKE 2 and DEMETER LIKE 3 prevent hypermethylation at specific genomic regions. These two are expressed in vegetative and reproductive tissue [200]. The ROS1 DNA demethylase has been identified as targeting particularly TEs in a genomic region closer to protein-coding genes, revealing activation of nearby genes through the demethylation process [201]. It is now recognized that short RNAs may also guide DNA methylation, a process known as the RNA-directed DNA methylation pathway (RdDM) through the activity of two kinds of RNA polymerases—PolIV and PolV [202]. A number of studies have shown that, when plants are exposed to heat stress, DNA methyltransferase (MET1, DRM2 and CMT3—which are the largest subunits of PolIV and PolV) is upregulated. The upregulation of these methyltransferases leads to more methylation under heat stress conditions in *Arabidopsis* [203].

It is well documented that modifications in histones are reversible, as the overlapping process of methylation and acetylation are very much important for stress response in plants. The acetylation process is governed by Histone acetyltransferase (HATs) and Histone deacetylases (HDACs) that help the plant adapt to changing environments [204]. In *Arabidopsis*, a total of 12 HAT genes represent four HAT families (GENERAL CONTROL NONDEREPRESSIBLE5 [GCN5]-like [GCN5/HISTONEACETYLTRANSFERASE OF THE GNAT FAMILY {HAG}1, 2, 3}, p300/CBP [CREB binding protein]-like [HISTONE ACETYLTRANSFERASE OF THE CBP FAMILY {HAC}1, 2, 4, 5, 12], TAFII250-like [HISTONE ACETYLTRANSFERASE OF THE TAFII250 FAMILY {HAF}1, 2] and, MYST-like [HISTONE ACETYLTRANSFERASE OF THE MYST FAMILY (HAM) 1, 2] [202,205]. For example, in soybean, the high salinity stress leads to methylation and histone modifications required for the activation/repression of stress-responsive TFs [206]. Salinity stress has a significant impact on genome-wide histone modifications and methylation for providing tolerance against such osmotic stress [204]. GCN5 was first characterized in maize root tissue under salt stress response. Up-regulation of cell-wall-related genes such as ZmXYLOGLUCAN ENDOTRANSGLUCOSYLASE/HYDROLASE1 and ZmEXPANSIN B2 are linked with acetylation of H3K9, which is a histone protein (H3) with lysine (K) on the 9th position, in both the promoter and coding region. Mutant *gcn5* exhibits salt sensitive response in maize because of the cell wall integrity [207]. In another study, *Arabidopsis* GCN5 plays an important role in heat tolerance through H3K9/k14 acetylation process in the promoter sequence of the ULTRAVIOLET HYPERSENSITIVE6 and, HEAT SHOCK TRANSCRIPTION FACTOR A3 genes [208]. Under cold stress, the C-REPEAT BINDING FACTOR (CBF)-COLD RESPONSIVE (COR) pathway augments to plant for survival. Induced expression of TFs, such as CBF family proteins under cold stress, bind over the COR gene promoter to facilitate its COR expression [209]. Heat shock proteins (HSPs) play a wide role in heat stress tolerance in plants. Accumulation of H3K4me3 and H3K9Ac has also been reported on HSP70, HSP22, HSP18 and APX2 [210]. Under different abiotic stresses, drought is well documented as a major abiotic stress factor that makes histone alterations [210,211]. NCED3 (NINE CISEPOXYCAROTENOID DIOXYGENASE 3) is a well-reported gene that responds to ABA synthesis under water scarcity conditions. Accumulation of H3K4me3 in NCED3 gene regions helps with drought resistance in plants accompanied by NCED3 gene expression [193]. Overall histone modification is a complicated process that plays an important role in epigenetic regulation. For example, histone H2A.Z is essential for the repression of unwanted transcription of drought-inducible genes’ expression in *Arabidopsis* [212]. On the other hand, H2A.Z is contributing to the grain yield of the *Brachypodium* under heat stress [213]. These contemporary data for H2A.Z suggest the diverse role of epigenetic-mediated gene regulation through structural divergence or PTMs of histone proteins that define a crucial topic to understand [213].

## 7. Metabolomics

**Figure 3:**
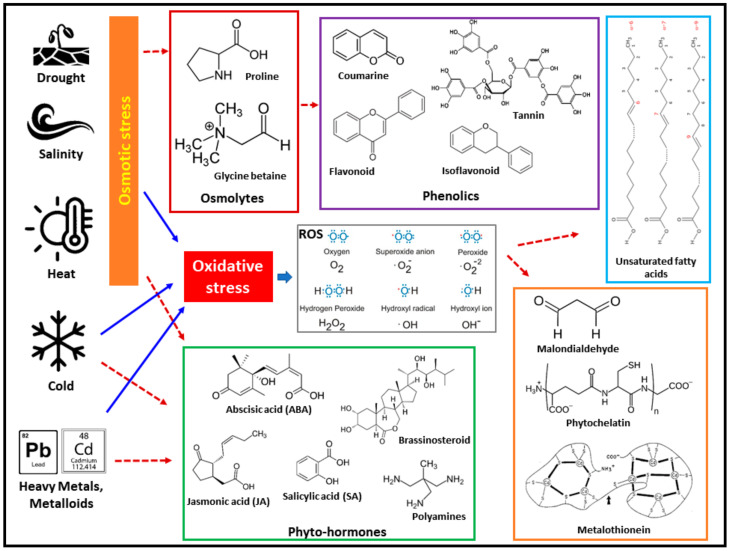
Involvement of different phytohormones, metabolites and other bioactive chemical components for abiotic stress response in plants.

Metabolites are concerned with the quantitative, qualitative and dynamic study of all endogenous, low molecular weight compounds (less than 1000–1500 dalton) within the organismal cells, tissue or organs and perform essential activities in a spatio-temporal manner. The plant kingdom contains approximately 0.2–1.0 million distinct metabolites whose concentrations vary from one species to another. These compounds vary from each other by their classes, physiochemical properties, chemical structure and polarity level [214]. A quantitative and qualitative study of plant metabolites responding to several environmental and biotic stress is not only a descriptive feature of plants but also reflects the genetic and biochemical background in stress-responding plants which brings the difference among plant species according to their level of tolerance and adaptation in particular stress [215,216]. Metabolomics has its advantage over the field of genomics, transcriptomics and proteomics. Metabolites are the downstream product of gene and protein activity that define the effect on living phenotype and other physiological activity [217,218,219]. Metabolites can be classified into two classes—primary and secondary metabolites. Primary metabolites are essential for plant growth and play a wide role in physiological activity [220], while secondary metabolites are essential for defence response under a wide range of abiotic stresses [221]. Plants have been studied for their ability to adapt to a variety of environmental conditions by looking at how they modify metabolites such as osmoprotectants (proline, glycine betaine, trehalose, etc.) and antioxidant enzymes (superoxide dismutase, peroxidase, ascorbate peroxidase, catalase, glutathione reductase, guaiacol peroxidase, etc.) (Figure 3) [222,223,224,225]. Primary metabolites such as sugars, amino acids and TCA (Krebs) cycle intermediates (citric acid, α-ketoglutarate) are directly involved in plants’ normal growth and development; whereas the secondary metabolites are genera-specific and condition-specific. Thus, the total metabolite profile of a given plant species indicates how many regulatory systems, such as gene expression and gene–protein interaction, have been integrated. Under any adverse environmental conditions, plants exhibit an array of responses that lead the particular stress tolerance, all of which are associated with metabolic modifications. Therefore, the study of stress-associated changes in metabolites is given particular attention in the 21st century [226,227]. Bioactive chemicals including antioxidants, signalling compounds, biosynthesis intermediates for cellular structures and storage compounds are produced when a metabolic pathway is activated. The production of these compounds, in turn, regulates or activates other compounds or intermediates that can feedback activate or inactivate different metabolic steps [228]. Polyamines are one of the well-reported secondary metabolites that contribute to plant growth and provide resistance under abiotic stress conditions in angiosperm plants. Spermidine (SPD), spermine (SPM) and putrescine (Put) are the common well-known polyamines found in almost all land plants. In cotton, the *Put* gene is expressed under the regulation of the *Arginine decarboxylase2* (*ADC2*) gene promoter in a salinity environment [229]. It has been reported that drought favors the accumulation of several secondary metabolites such as alkaloids, terpenes and complex phenols. For example, drought stress induces the phenolic content in *Hypericum polyanthemum* (hypericum)*, Salvia officinalis* (garden sage)*,* rice and barley. Likewise, the monoterpenes or terpenoids amount also increases in Barley and *Salvia officinalis* under drought stress [230,231,232]. Similarly, trehalose, which is a non-reducing disaccharide, plays a beneficial role to maintain membrane integrity and stabilization of macromolecules under drought conditions. The rate of photosynthesis is also increased under overexpression of trehalose and PSII is protected against photo-oxidation through trehaloses [233]. Variation of the polyamines profile has been observed in salt-tolerant rice and tomato species under diverse stress conditions [234]. Heat stress also induces the overproduction of flavonoids, phenylpropanoids and phenolic metabolites through the upregulated overexpression of the associated genes [235]. In leaves of tomatoes, heat stress factor: HsfB1 suppression or overexpression increases thermo-tolerance capacity in the plant. The overexpression of HsfB1 leads to the accumulation of phenylpropanoid products and the pathway of flavonoid in addition to various isomers of caffeoyl quinic acid [236]. In an aspect of salinity stress, omeprazole (proton pump inhibitor) helps to enhance the salt stress tolerance in tomato and makes several hormonal changes in leaves such as abscisic acid increment while decrementing auxin, cytokinin and gibberellic acid levels. Additionally, alkaloids and sesquiterpenes are conjugated with polyamines under the response of Omeprazole [237]. Under oxidative stress, ROS are overproduced in the plants, causing oxidative deterioration of cellular macromolecular structures such as DNA, RNA, protein and lipids [238]. To overcome this ROS-mediated oxidative stress, a powerful ROS scavenger, glutathione, is an essential antioxidative metabolite [239]. Various other metabolites such as proline, polyphenols, ascorbic acid, carotenoids and tocopherols act as nonenzymatic antioxidant molecules [240]. Under salinity stress, the cellular antioxidant level is increased [241]. The ascorbate–glutathione cycle is an important biochemical pathway as well as the potent non-enzymatic antioxidant system that is used to detoxify several toxic compounds and ROS in living cells generated under abiotic stresses. The ascorbate–glutathione pathway detoxifies the methylglyoxal (MG), which is a highly reactive cytotoxic compound. MG is accumulated under adverse abiotic stress conditions [242]. Several other primary metabolites such as organic acids have their importance for different abiotic stress. For example, malic acid provides drought resistance in different plant species such as tropical grasses, cotton and spare grasses [243]. Overexpression of galacturonic acid reductase in potato genotypes helps to increase the content of ascorbic acid and water stress tolerance [244].

Alteration in many metabolic pathways in plant cells and organs contributes to the balance of the metabolite profile in the organism. Presently, several detections and analytical separation techniques are used in combination for the visualization of an organism’s metabolomic profile. With the advancement in MS, nuclear magnetic resonance (NMR) and chromatographic techniques, a large number of metabolite analyses and studies have become quite handy for scientists [245,246]. Ghatak et al. [246] provide detailed information about plant metabolomic methods, libraries used in the analysis, data mining and processing, chemical identification and the limits of metabolomics. LC-MS/MS and gas chromatography-MS (GC-MS) are popular techniques for metabolomics study because of their unparalleled level of sensitivity and extensive coverage of huge metabolites [136]. Development of new analytical techniques such as GC, LC coupled to MS, NMR, Fourier Transform Infrared spectroscopy (FTIR) or capillary electrophoresis (CE) provides a more accurate description of metabolite interactions in a given plant species [247]. Characteristics of several stress-responsive metabolites can be detected and quantified concurrently using mass-spectrometry-based metabolomics methods [248]. However, the molecular heterogeneity and broad-spectrum metabolome significantly hamper compound identification and meaningful measurement. Ion suppression, fragmentation and the existence of isomers may further complicate the simultaneous measurement of multiple metabolites within complex phytochemical mixtures. To facilitate high-quality reporting of data derived from LC and GC-MS-based metabolomics, Alseekh et al. [248] recommended encompassing the preparation of samples, reproduction and randomization, quantization, restoration and recombination, ion suppression and identifying incorrect peaks. Moreover, a few more techniques such as targeted analysis, metabolic fingerprinting and metabolite profiling are utilized for speed, improved comprehensiveness, better resolution, the throughput of analytical assays and miniaturization equipment [249]. Meanwhile the combination of several techniques such as LC-MS/MS, CE-MS/MS, ultra-high performance liquid chromatography-quadrupole time-of-flight mass spectrometry (UHPLC-TOF-MS) or nuclear magnetic resonance [such as LC-NMR or LC with PhotoDiode Array Detection-Solid phase extraction-NMR-MS/MS (LC- DAD-SPE-NMR-MS/MS)] joint with bioinformatics tool is of great assistance for the study of the natural products of plants and clears the vision of comprehensive profiles of metabolites [165,250].

## 8. Proteogenomics, Lipidomics, Ionomics and Interactomics

Proteogenomics is a new and integrative approach that combines the technological advancement of genomics, transcriptomics and proteomics together [251]. The goal of typical proteogenomics research is to catalogue the proteins that are already being expressed in the cell by combining high-throughput NGS data with MS-based proteomics applications [252]. Particularly, proteogenomics is useful for the identification of proteins by integrating genomic, transcriptomic and MS data of the same crop species and/or sample. Thus, the integrative proteogenomics approach has identified novel proteins and provided a hardcore knowledge of the genes’ regulatory expression and cell signalling for abiotic stress tolerance [253]. Proteogenomics has shed light on the mechanisms of plants’ response to abiotic stress and adaptation to changing the environment. Phosphorylation of protein molecules is to be considered in the proteogenomics phenomenon [254]. Under salinity stress, the primary and secondary transporters depend upon phosphorylation for being active and to regulate the sodium ion (Na^+^) but, still, the information on responsive kinases is not explored much for most economically important crops [255].

Lipidome describes the whole profile of lipids in the cellular, tissue or organ level of an organism. Lipidomics is an emerging field of science that studies the structure and function of the lipidome as well as their interactions with other lipids, proteins and metabolites [256]. Lipids play a dual role in plants’ abiotic stress response. Besides being important signalling mediators, lipid molecules play significant roles in the alleviation of stress [257]. Signalling lipids such as fatty acids, sphingolipids, diacylglycerols, lysophospholipids, phosphatidic acid, inositol phosphate, oxylipins and N-acylethanolamine are quickly synthesized under stress conditions. Simultaneously, lipids are also involved in the remodelling of cell membranes under abiotic stress and mitigate cell damage [258]. Improvement of analytical methods, particularly liquid chromatography and mass spectrometry, enables systems-level analysis of lipids and their interacting partners [259]. The tools of lipidomics are categorized into two broad categories—MS prediction tools and structure-drawing tools [260]. The MS-based methods have been shown to be highly efficient for the characterization and quantification of molecular lipids. The different MS techniques are categorized into three groups—global lipidomic analysis (GLA), targeted lipidomics analysis (TLA) and novel lipid discovery (NLD). The GLA is a high-throughput method of identification and quantification of cellular lipid species. It is particularly useful to analyze and decipher pathways and networks associated with lipid metabolism, trafficking and homeostasis [261]. TLA employed LC-MS and LC-MS/MS based on the identification of lipid molecules, whereas NLD uses LC coupled with MS and is involved in the finding of novel lipid species. Methods such as MALDI-TOF MS coupled with thin-layer chromatography (TLC) are presently being used for imaging lipids from tissue slides [262]. A particular lipid profile of a given crop species under certain abiotic stress conditions can act as a lipid biomarker or lipidotype. Sun et al. [263] identified the leaf lipid profile in Begonia and its alterations under heat stress, which helps to understand the stress adaptive mechanism in plants. The lipidomics process may be paired with MS-based methods and robust GWAS (lipidomics-aided GWAS or LiA-GWAS) to find membrane lipid remodelling-associated genes and likely relationships that may be exploited to generate stress-tolerant plants [256]. However, because of its complexity and specificity, lipidomics studies are complicated and quite challenging. Moreover, due to the great diversity in lipid classes, the structural identification of lipids is a complicated process. Hopefully, in the near future, the development of comprehensive lipidomics technologies developed will expand the sphere of plant lipidomics and shed more light on the involvement of lipid molecules during abiotic stresses.

In ionomics, elements are profiled in a high-throughput manner and deal with the studies of inorganic components, mineral nutrients and trace element composition of a living being [264]. Genomic data aided with ionomics, particularly in combinations with forward and reverse genetic approaches, can detect cellular changes during abiotic stress conditions [265]. Recent reports showed that ionomics studies revealed the mechanisms of ion uptake under abiotic stresses in plants [266,267,268]. Moreover, transport, compartmentalization and exclusion of ions during adverse environmental conations were also monitored by ionomics. Although ionomics is a new field and there are only a few reports of ionomics studies under abiotic stress available, the trends are growing for ionomics studies [266,267,268]. In a general sense, ionomics are related to the ion content of an organism (here in plants) that is required for its growth and developmental processes under different environments and growth stages [269]. In plants, ions are classified under two major categories: essential nutrient ions (macro and micronutrients) and non-essential nutrient ions [270]. Apart from this, some of the non-essential ions harm normal physiological conditions in the plant. For example, the sodium ion (Na^+^) is a well-reported causal factor of salinity stress in many glycophytes, including rice, in which the productivity of grain yield is drastically affected [271]. The role of ion transport regarding how Na^+^ is regulated by primary and secondary transporter under salt stress in both tolerant and sensitive rice lines has been identified [151]. There is also a positive role of an essential ion such as Ca^2+^ that antagonizes the salt stress (Na^+^) effect in rice salt-tolerant landraces Nona Bokra and sensitive cultivar IR-64 [272]. In terms of essential ions, phosphorus (P) is one of the most important ions that help in major biological activities in the plant system [273]. The importance of phosphorus nutrients has been revealed in EMS-induced mutants compared to Nagina N-22 rice under low and normal P soil conditions and could have accounted for improved physiological and biochemical activity under low P field conditions [274].

Interactomics is the study of the interactions and the consequences of those interactions among the biomolecules in a cell [275]. Complex physical, biochemical and functional interactions between DNA, RNA, proteins, lipids and tiny metabolites mediate cellular processes. The term interactome most usually refers to a network of protein–protein interactions (PPIN) [276]. However, another important interactome is the protein–DNA interactome which is also known as the gene-regulatory network. Thus, the plant interactome constitutes TFs factors and chromatin regulatory proteins with the genes of their target site [277]. Since protein–protein and protein–DNA interactions are central to all cellular processes, understanding these interactions in both normal and stress conditions facilitate the identification of underneath regulatory mechanism of stress tolerance. In recent years, many different technologies have been developed for interactomics study. All these technological approaches are broadly categorized into three heads: in silico, in vivo and in vitro. The in silico methods are carried out by computer simulation and consist of text mining and computational analyses. The in vivo methods are performed on intact living individuals. The yeast two-hybrid (Y2H), protein-fragment complementation assay (PFCA) and protein–protein interaction trap (MAPPIT) are common in vivo approaches to interactomics. The experiments of in vitro methods are performed outside a living organism and under controlled conditions. The in vitro approaches of interactomics include techniques such as tandem affinity purification-MS (TAP-MS), protein microarray and the luminescence-based mammalian interactome (LUMIER) tools [278]. Though there are few instances of involvement of interactomics in plants for abiotic stress tolerance, its proper high-throughput applications are still underway [279,280].

## 9. Phenomics

**Figure 4:**
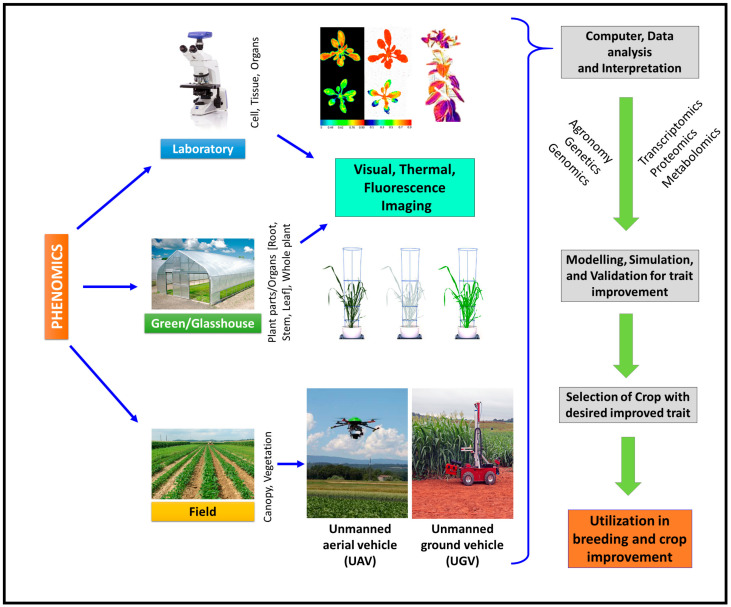
Phenomics platforms are represented schematically to assess agricultural productivity for abiotic stress-responsive future breeding.

Phenomics deals with omics of phene (phenotypes), a product of genes and utilizes high-throughput analysis of organismal phenotype by evaluating the morphological, physiological, and biochemical traits [281]. In the case of plants, phenomics correlates growth, performance and composition with genetic, epigenetic and environmental factors. Therefore, phenomics integrated with other omics unveils cellular biochemical or bio-physical networks that result in the final desirable phenotype [282]. As most phenotypic traits are determined by the interactions between genes and the environment (G × E), collections of large numbers of phenotypic data across multiple environmental conditions revealed the relationships between phenotypic traits and prevailing abiotic stresses [283,284]. Both forward and reverse phenomics strategies were employed for the analysis of various traits. Forward phenomics uses high-throughput and fully automated phenotyping tools for the rapid identification of interesting, unique or desired traits [285], whereas the reverse phenomics method investigates the selected traits in detail and subsequently discovers the underneath mechanism [286]. In fact, phenomics can be used at the cellular and tissue level and also used on a bigger scale, i.e., plant organ, whole plant, plant community in the field, the vegetation of the particular area and ecosystem basis (Figure 4).

In recent years, for large-scale precise, accurate and rapid trait phenotyping, high-throughput non-invasive imaging technologies became quite popular [287,288,289]. High-throughput phenotyping (HTP) estimates the quantification of chlorophyll fluorescence, the water content in the leaf and other associated plant parts and geographical parameters [290]. HTP encompasses image-based techniques such as visible light imaging, fluorescence imaging, hyperspectral imaging, infrared (IR) imaging and X-ray computed tomography, and these techniques are controlled by a robust software system [291]. Image-based automated HTP integrates advanced software that is feasible to access for plant biology research [292]. Visible light imaging techniques are based upon a two-dimensional (2D) digital imaging system to measure leaf morphology, canopy coverage, above-ground dry matter, seed and panicle morphology, the architecture of root, shoot tip extension and yield-associated traits [293,294]. Apart from the 2D imaging system, 3D imaging techniques have also been reported to measure different characteristics in plants, such as leaf morphology, plant height, above-ground dry matter, crop structure and stature [295]. Plant eye is a 3D-based imaging technique that has been reported to observe the area of the leaf with the wet (fresh) and dry matter in wheat under salinity stress [296]. A study of a photosynthetic function under abiotic stresses by Chlorophyll Fluorescence Analysis (CFA) can discriminate between susceptible and tolerant genotypes [297]. Fluorescence Imaging is also widely used for detecting the stress in plants system at the primary level [298], which becomes helpful to resolve the heterogeneity in photosynthetic performance based on chlorophyll fluorescence in the leaf [299]. Likewise, the Chl F transient (Chlorophyll fluorescence) technique is used to discriminate the cold-sensitive and tolerant *A. thaliana* species [300]. Pulse Amplitude Modulated (PAM) or fluorometry can measure fluorescence parameters in plants [301] and is successfully used for screening *Arabidopsis*, tobacco and cotton (*Gossypium* ssp.) [302,303,304].

Recently, leaf spectroscopy, hyperspectral reflectance spectroscopy and imaging sensors related to chlorophyll fluorescence have also been successfully used for the study of phenomics under abiotic-stress conditions [305]. Digital imaging is a popular method of in situ plant phenotyping. In the last decade, numerous techniques and methodologies have been developed for automated phenotyping [306,307], which will provide valuable information about the abiotic stress tolerance of plants. PlantDIP (plant digital image processing) related to Scanalyzer HTS has been demonstrated to estimate the high ascorbic acid (vitamin C) content for osmotic stress response in *Arabidopsis* model [308]. Red, green, and blue color-based phenotyping (RGB phenotyping) are accounted for the measurement in various crops under abiotic stress conditions with the parallel use of computational software such as WIWAM (https://www.wiwam.be/ accessed on 6 March 2023) and PHENOPSIS [309]. Similarly, Lemna Tech is reported to use in barley and maize for drought stress [310,311,312] and in rice and wheat for salinity [313,314]. Li-Cor 6400 is a modern noninvasive HTP tool that has been considered for leaf gas exchange parameter study as reported in grapevine under drought stress [315]. It is widely used to study physiological parameters such as intercellular CO_2_, transpiration rate, stomatal conductance and photosynthetic rate under different abiotic stress environmental conditions [316]. The application of spectroscopy imaging is widely used for field phenotyping through aerial platforms. Hyperspectral high-spatial resolution satellite data are very effective in analyzing the physical and empirical analysis of water content in the canopy [317]. Unmanned aerial vehicle (UAV) is a thermal-based remote sensing noninvasive method that has accounted for drought stress response in poplar plants [318]. Apart from the drought stress, UAV-based HTP also accounted for salinity stress response in wild-type tomatoes and date palms [319,320]. In addition, the heat stress effect on crops and lodging should be accounted for. Common indicators for agricultural plants include the vegetation indices (VIs)—normalized difference vegetation index (NDVI), excessive green (ExG) and green leaf index (GLI), all of which represent canopy features and crop phenology that are significantly influenced by stress situations [321,322]. NDVI is a graphical form of obtained remote sensing data that are widely used in crop phenotyping [323]. Its calculation is based on the reflectance spectrum under red and near-infrared (NIR) regions of light in the plant [28]. It is one of the phenotypic tools that can be used for the estimation of canopy temperature and height and has high applicability to analyze heat, drought, water and salinity stress [324,325,326,327]. The ExG index is one of the parameters (based on RGB images) that has been reported for crop water stress index (CWSI) as well as water potential of leaf analysis in maize canopy [328]. Forster resonance energy transfer (FRET) is another advanced noninvasive method that has been applied to track the zinc and calcium dynamics in the root tissue during the transport of sugar [329]. Positron emission tomography (PET) is also reported to measure the stress effect on photosynthetic performance [292]. Infrared thermography has been reported to study stomatal activity under drought and salinity stresses through differences in plant canopy temperature and structure [330]. Infrared-image-based techniques provide high-quality measurements with high spatial resolution images under a broad range of climatic conditions [331]. Temperature differences of the canopy among different plant species can be applied for drought stress tolerance under dry (arid and semi-arid) environmental situations. The thermal-based infrared imaging system is widely performed in laboratory and field conditions to characterize drought, salinity and heat stress based on Na^+^ exclusion and osmotic imbalance [332]. With the application of infrared imaging, it is feasible to detect the significant differences among leaf, canopy and environmental temperature under high temperature and drought stress which is well reported in fruits and vegetables [292,327,328,329,330].

## 10. Integration of Multi-Omics Data and Interpretation for Abiotic Stress Response in Plants

**Figure 5:**
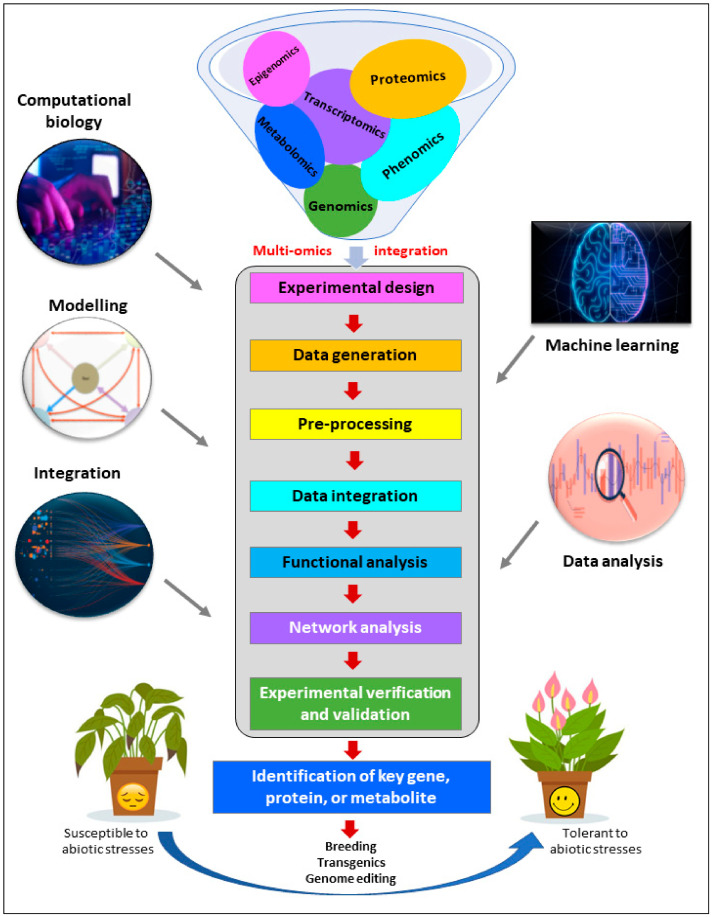
Integrated multi-omics pipeline for abiotic stress tolerance response in plants.

Integrating multi-omics methods is basically to connect genotype to phenotype for a proper understanding of the biological processes such as abiotic stress response in plants. Multi-omics data integration is a powerful approach that combines information from multiple high-throughput omics technologies, such as genomics, transcriptomics, proteomics, metabolomics, epigenomics, etc., to gain a comprehensive understanding of complex biological systems (Figure 5). This approach has been widely used in diverse crops to investigate the molecular mechanisms underlying abiotic stress tolerance responses which are needed to make tolerant crop varieties [333]. Integrating multi-omics data can provide a more holistic view of how plants respond to abiotic stresses at the molecular level. Here a general overview of the steps involved in multi-omics data integration is represented for studying crop abiotic stress tolerance responses.

‘Experimental design’ is the first step to planning and designing experiments that expose crops to specific abiotic stress conditions while considering appropriate control sets for a quick compare. It needs to ensure the collection of samples for multiple omics platforms, including DNA, RNA, proteins and metabolites. ‘Data generation’ utilizes high-throughput omics technologies such as whole genome sequencing (WGS) for genomics, RNAseq for transcriptomics, MS for proteomics and metabolomics profiling for metabolomics, to generate large-scale datasets for each omics layer [334]. ‘Data pre-processing’ performs quality control and pre-processing steps specific to each omics platform. This may involve read-trimming and alignment for genomics and transcriptomics data, quality control (QC), data normalization and missing value imputation for proteomics and metabolomics data and removing batch effects if multiple experiments are involved. ‘Data integration’ is a very vital step which can apply computational methods to integrate the multi-omics datasets. Such big-data-driven analysis requires high statistical significance to integrate different omics layers. For easy visualization and analysis, these interconnections are analyzed using functional and statistical networks to validate results obtained by multi-omics layers. Different strategies can be employed, including correlation-based approaches, network-based approaches and machine learning (ML) algorithms. PaintOmics 4 is a new web-based server to integrate multi-omics datasets using biological pathway maps [335]. It is crucial in data integration to combine data from several sources in order to build a model that can be used to predict complicated features and increase prediction accuracy. In order to predict phenotypes, an increasing variety of statistical models, including both linear and nonlinear models, have been created and are currently in use [336]. Several linear models, such as Genomic Best Linear Unbiased Prediction (GBLUP), Linear mixed models (LMMs), Bayesian sparse linear mixed model (BSLMM) and Penalized linear mixed model with generalized method of moments estimator (MpLMMGMM) model, are widely used to model multi-omics data with higher phenotypic prediction [336]. On the other hand, ML is one of the nonlinear methods that use both supervised and unsupervised learning programming paradigms with statistical inference from big complex data. Two main objectives to be predicted in supervised learning are categorization and regression. Unsupervised learning is frequently employed to look for data interpretations including grouping, association and dimensionality reduction (DR) which is significant in high spatial biology since it minimizes the number of random variables to take into account [337]. These methods aim to identify relationships and interactions between molecules across different omics layers. ‘Functional analysis’ can interpret the integrated multi-omics data to gain insights into the molecular mechanisms underlying abiotic stress tolerance responses and may involve GO analysis, pathway enrichment analysis and functional annotation of key genes, proteins and metabolites [23]. ‘Network analysis’ is to construct biological networks that capture the interactions between different molecules identified in the integrated data. Network analysis techniques, such as co-expression networks or protein–protein interaction networks, can help identify key hub genes or proteins involved in stress response [338]. In the end, ‘experimental verification and validation’ involve the selection of candidate genes, proteins or metabolites identified from the integrated analysis for experimental validation. Techniques such as qPCR, Western blotting or targeted metabolomics can be used to validate the findings and confirm their roles in abiotic stress tolerance. This approach is also necessary to validate data and reveal post-transcriptional and post-translational mechanisms of gene expression regulation [339].

Omics-integration is much more positive when it can apply to early plant life, i.e., at the seedling stage. It has validated the possibility of applying a non-targeted integration approach to non-model plant *Quercus ilex* for early response to drought [340]. Transcriptomics, proteomics and metabolomics data use two integrative approaches, Principal Component Analysis (PCA) and Data Integration Analysis for Biomarker discovery using Latent variable approaches for Omics studies (DIABLO), which permits interconnections between the different omics-layers to be inferred and enables the discovery of key processes such as transcriptional control and to identify the key function TFs [340]. Multi-omics integration was also evident in oil palm for drought and salinity response by applying transcriptomic, proteomics and metabolomics [341,342]. Differential enzymes and metabolites identified from the analysis highly correlate (r ≥ 90) with cysteine and methionine metabolism pathways affected by the osmotic stress [341,342]. Integration of root multi-omics (transcriptomics, proteomics and metabolomics) reveals drought stress tolerance response in chickpeas. Integration of transcriptomics and proteomics data was able to identify enriched proteins hubs and integration of root-omics data also revealed some key candidate genes underlying drought-responsive ‘QTL-hotspot’ [343]. By integrating multi-omics data, a deeper understanding of the regulatory networks and molecular mechanisms governing crop responses to abiotic stress is possible. This knowledge can be leveraged for the development of stress-tolerant crop varieties through targeted breeding and genetic engineering (transgenic and genome editing) strategies, as well as for the identification of potential biomarkers or targets for future crop development.

## 11. Conclusions and Perspectives

Different existing omics approaches are overlapping and are interconnected with each other; and allow the identification of integrated cellular activities leading to stress responses and tolerance levels of a plant. To conclude and fully understand the primary cell response cascades that may vary between tolerant and sensitive plants under certain abiotic stress conditions, it is essential to integrate multi-omics data gathered through various omics pipelines. After revealing the crop’s response through multi-omics-aided non-DNA markers such as transcripts, proteins, metabolites, etc., those crops can be used as important genetic resources and incorporated into the breeding and genetic engineering strategies for making stress-tolerant plants. Here, we have broadly reviewed diverse multi-omics approaches for studying stress response and adaptive mechanisms of a plant under abiotic stress conditions. For the practical utility in breeding, we may consider marker assisted breeding (MAB) or its advanced version—genome assisted breeding (GAB) for plant’s abiotic stress tolerance, but these are only dealing with genomics. However, multi-omics and omics integration facilitate to open a new avenue of ‘Omics-assisted breeding’ which can also utilize GAB to enhance crop yield, quality attributes and other associated agronomic parameters along with the particular abiotic stress tolerance. Multi-omics-based analysis can integrate data from various omics platforms and provide a comprehensive systems-level understanding of abiotic stress tolerance in crops, offering the identification of key regulatory networks, biomarkers and candidate genes that can be targeted for breeding efforts, enabling precision agriculture strategies. Such multi-omics integration output can be a reliable strategy for linking genotype by the phenotype of a plant. This holistic approach increases the chances of success in developing stress-tolerant crop varieties. The big data obtained from the multi-omics layers, combined with advanced bioinformatics and computational tools, can be used for predictive modelling and precision breeding by applying machine learning algorithms. These achievements contribute to the development of stress-tolerant crop varieties and sustainable agricultural practices, ensuring food security in the face of changing environmental conditions.

## Figures and Tables

**Table 2:** Important genes involved in plants’ responses to abiotic stresses.

Plants	Genes	Function	Abiotic Stresses	Note/Remarks	References
Barley	*rhl1.a*	Limit root hair	Drought	Loss of function mutation	[114]
Maize	*Zm00001d010956*	Induce TFs	Salinity		[123]
Tomato	*SlERF.D6*	Steroidal glycoalkaloids (SGAs) biosynthesis	Drought, Salinity		[129]
Alfalfa	*HAMK*	Signaling pathway	Heat		[135]
Bread wheat	*HVA1*	Signaling pathway	Drought, Heat	*Hordeum vulgare* aleurone 1	[136]
Wheat	*AtWRKY30*	TFs	Heat, Drought	Antioxidant, osmolytes biosynthesis	[137]
Arabidopsis	*AtSZF2*	Regulate salt-responsive genes	Salinity	Loss of function mutation	[138]
Arabidopsis	*CBF1*	TFs	Cold, Heat	C-Repeat Binding Factor1	[139]
*Arabidopsis*	*PICKLE (PKL)*	CHD3-type chromatin remodeler	Cold	Trimethylation of histone H3 lysine 27 (H3K27me3)	[140]
Tomato	*SlAREB1*	TFs	Salinity		[141]
Tomato	Ethylene Response Factors *(ERF)*	TFs	Cold, Heat, Salinity, Drought, Submergence		[142]
Tomato	*SlUVR8*	UV-B photoreceptor	UV-B		[143]
Wild tomato (*Solanum habrochaites*)	Calmodulin-like *(ShCML44)*	Calmodulin-like (CML) proteins Ca^2+^ sensors	Cold, Drought, Salinity	Enhances Antioxidants Capacity	[144]
Cabbage	*HSP70*	Heat Shock Protein	Heat		[145]
Pearl millet	*PgDREB2A*	Transcription Factor	Heat, Drought, Salinity	Transgenic overexpression in tobacco	[146]
Rice	*OsMAPK5*	Signaling pathway	Drought, Salinity, Cold		[147]
Rice	*OsWRKY87*	Increase DNA-binding ability	Drought, Salinity		[148]
Rice	PM H^+^ ATPase	Proton pump	Salinity	Primary transporter	[149]
Rice	Cellulose synthase-like protein *OsCSLD4*	Cell wall polysaccharide synthesis	Salinity	ABA-induced osmotic response	[150]
Rice	Sucrose non-fermenting 1-related protein kinases *(OsSAPK8)*	Serine/threonine (Ser/Thr) protein kinase	Cold, Drought, Salinity		[151]
Soybean	*MAPK*	Signaling pathway	Excess light		[152]
Ground nut	*NAC4*	TFs	Drought		[153]
Ground nut	*HSP70*	Chaperon	Heat	Heat shock protein	[153]
Potato	*LEA*	Cellular protection during stress	Drought, Salinity, Heat	Late Embryogenesis-Abundant protein	[154]

## Data Availability

Not applicable.
